# Generative AI in psychiatric education: balancing risks and benefits for responsible integration

**DOI:** 10.3389/fpsyt.2026.1894299

**Published:** 2026-07-29

**Authors:** Cong Zhou, Aoxue Zhang, Sen Li

**Affiliations:** 1 School of Mental Health, Jining Medical University, Jining, China; 2 Jining Key Laboratory of AI-Assisted Diagnosis and Treatment of Mental Disorders, Jining, China; 3 Department of Psychology, Affiliated Hospital of Jining Medical University, Jining Medical University, Jining, China

**Keywords:** ethics, generative AI, medical education, mental health, psychiatric education

## Abstract

Generative artificial intelligence (GenAI) chatbots, including ChatGPT and DeepSeek, are rapidly reshaping psychiatric education across the continuum from medical students to practicing psychiatrists. These tools offer learners unprecedented opportunities for personalized learning, clinical simulation, and research support. However, their integration also raises substantial risks related to data privacy, content reliability, over reliance on automation, and ethical and regulatory gaps. This perspective argues that neither uncritical adoption nor outright rejection is appropriate. Drawing on emerging evidence and our firsthand experience as psychiatric educators, we propose a balanced framework characterized by three interconnected principles: strong human oversight, comprehensive AI literacy training, and robust institutional governance. We outline five categories of risk, discuss potential benefits specific to psychiatric training, and offer six actionable institutional recommendations. The goal is not to deter innovation but to ensure that GenAI tools enhance rather than replace the clinical judgment, empathy, and professional accountability that lie at the heart of psychiatric practice.

## 1 Introduction

The rapid emergence of generative artificial intelligence (GenAI) and large language models (LLMs) has begun to transform psychiatric education in ways that educators can no longer afford to ignore. Unlike previous educational technologies that primarily delivered static content, GenAI offers dynamic, conversational, and increasingly sophisticated interaction. It can simulate clinical encounters, generate personalized study materials, and provide real time feedback on clinical reasoning (1).

A scoping review by Lee et al. found that GenAI has significant potential to enhance psychiatric education, particularly in areas such as clinical simulation and personalized content delivery (2). However, the same review noted that limitations require careful management. Another scoping review on AI applications in psychiatry and psychology education concluded that AI is being used across a range of educational functions, from clinical training to assessment and administrative support, but the evidence base remains fragmented and preliminary (3). Omar et al. identified diverse applications of LLMs in clinical reasoning, social media, and education within psychiatry, while stressing the need for rigorous validation (4). A more comprehensive scoping review by Weightman et al. (2025) examined 26 records published between 2016 and 2024, identifying six key themes including the imperative for an AI curriculum, uses of AI for educational resource development and clinical skills training, AI in assessments, academic integrity and ethical considerations, and tensions relating to competing priorities (5).

As psychiatric educators, we face a central paradox. How can we harness these powerful tools without eroding the very competencies that define our profession, including clinical judgment, empathy, and professional accountability? The arrival of GenAI in psychiatric education is more than a technological update; it forces a fundamental reckoning. Educators and learners must now navigate a landscape filled with both unprecedented opportunities and significant attendant risks.

In this perspective article, we draw upon the emerging literature and our own experience as educators at a university school of mental health. We offer a balanced, pragmatic framework for integrating GenAI chatbots into psychiatric education. We argue for a stance that rejects both uncritical adoption and outright prohibition. Instead, we advocate for mindful integration guided by three core principles: (1) human oversight as an inviolable requirement, (2) AI literacy as a core curricular competency, and (3) institutional governance as a necessary safeguard. We use the term “psychiatric learners” to encompass the full spectrum of trainees in psychiatric education, from medical students during their psychiatry clerkships to residents and fellows in specialty training, with adaptations appropriate to each stage.

## 2 Why psychiatric education is uniquely vulnerable and potentially transformed

The deep integration of AI into psychological research and practice has been systematically documented in a recent bibliometric analysis. Jia et al. mapped the entire field of “AI-empowered psychology, “ identifying eight thematic domains including Computational Psychiatry and Digital Mental Health Intervention, and demonstrating AI’s dual function as both a research tool and an object of study (6). Psychiatry differs from other medical specialties in ways that amplify both the potential benefits and risks of GenAI. The core competencies of psychiatric practice include building therapeutic alliances, conducting nuanced diagnostic interviews, managing countertransference, and navigating complex ethical dilemmas. These skills are fundamentally relational and context dependent. They are precisely the skills most difficult to teach through traditional didactic methods and most vulnerable to erosion through over reliance on automated systems (7, 8). Unlike many other medical specialties where diagnosis is primarily pattern recognition based on objective tests, psychiatry places greater emphasis on formulation, the integration of biological, psychological, and social factors into a coherent understanding of the patient’s unique presentation. This narrative, contextual approach is a defining feature of psychiatric practice and is precisely the type of reasoning most difficult to automate or teach through AI.

In our experience as educators at a major psychiatric training institution, learners across all stages of psychiatric education are increasingly incorporating GenAI chatbots into their study and clinical preparation routines. This observation is consistent with recent survey findings from Chinese medical postgraduates, where 82.9% reported strong AI awareness and common applications included literature review, exam preparation, and clinical case analysis (9), though formal evidence quantifying these patterns across all stages of training remains limited. Even practicing psychiatrists may consult these tools for updates on clinical guidelines (7). The appeal of AI tools lies in their potential to offer several distinct advantages tailored to the demanding nature of psychiatric training. These include round the clock availability, which is particularly beneficial given the often-irregular schedules of medical learners. The rapid response capability of these chatbots to complex clinical queries can significantly aid in mastering intricate knowledge, understanding differential diagnoses, and clarifying sophisticated treatment algorithms (10). Medical students and residents differ in how they use GenAI. Medical students typically rely on these tools for foundational learning, including understanding basic psychopathology, diagnostic criteria, and exam preparation, during their brief psychiatry rotations, and they lack the clinical experience to independently evaluate AI outputs. Residents, by contrast, use GenAI more for clinical decision support, such as generating differential diagnoses and exploring treatment options, and they have greater clinical responsibility and more opportunities for reflective practice. These differences require stage-specific approaches to AI literacy training and oversight.

At the same time, precisely because these competencies require extensive practice and reflective feedback, GenAI offers unique affordances. AI powered conversational agents can simulate standardized patients with remarkable fidelity, as demonstrated in recent feasibility studies where generative pretrained transformer (GPT)–powered chatbots effectively simulated patients for clinical history-taking exercises (11), allowing learners to practice high stakes scenarios such as suicide risk assessment or breaking bad news in a safe, repeatable environment. The review by Ajluni highlighted that AI powered simulations can enhance diagnostic accuracy and clinical skill development while providing training opportunities that might otherwise be logistically or ethically prohibitive (12).

Beyond simulation, GenAI may offer support for certain aspects of research proficiency, such as accelerating literature synthesis and assisting with study design under appropriate supervision (1). However, we acknowledge that this is an area of active debate. Hough et al. (2025) have highlighted significant risks including inaccuracy, overreliance on AI-generated content, challenges to authenticity and academic integrity, potential biases in AI outputs, and privacy concerns (13). Some studies have raised concerns that over-reliance on AI may undermine the development of independent research skills by preventing students from engaging deeply with the research process. We therefore emphasize that any use of GenAI for research purposes should be carefully supervised and integrated with traditional research training. For learners experiencing burnout or psychological distress, a well-documented problem in medical education, AI chatbots may offer accessible, non-judgmental mental health support (14, 15). However, this application remains particularly contentious given current reliability concerns (16, 17).

As we discuss below, these potential benefits cannot be decoupled from substantial risks that are especially acute in the psychiatric context (18).

## 3 Five categories of risk

### 3.1 Data privacy and confidentiality

Psychiatry learners frequently interact with highly sensitive patient information. When such data is input into third party AI platforms, risks of re identification, data breaches, and non-compliance with regulations such as Health Insurance Portability and Accountability Act (HIPAA) become acute (19, 20). Many GenAI tools are cloud based, and their data handling practices lack transparency. This creates potential violations of both legal obligations and professional ethical codes. Research has documented that data privacy remains one of the most pressing concerns among clinicians regarding AI integration in mental health care (14).

### 3.2 Accuracy and reliability

LLMs are known to produce “hallucinations”, which are plausible sounding but factually incorrect or fabricated information (8, 18, 21, 22). While these errors pose risks across all medical specialties, they are particularly dangerous in psychiatry. Unlike fields that rely heavily on objective markers such as imaging, laboratory values, or clearly defined physiological parameters, psychiatric assessment relies heavily on narrative interpretation and clinical judgment, with few objective markers to detect inaccuracies (23). AI hallucinations in psychiatry may therefore go undetected longer and have more direct and serious consequences for vulnerable patients (24). For psychiatric education, this poses particular dangers. An AI generated summary of diagnostic criteria for a rare disorder might omit critical distinctions. An AI simulated patient might model atypical presentations as if they were prototypical. Research has shown that ChatGPT is not yet ready for use in providing mental health assessment and interventions (16). Without rigorous verification, learners risk internalizing incorrect information or developing flawed clinical schemas (25, 26).

### 3.3 Over reliance and erosion of clinical judgment

Perhaps the most insidious risk is the gradual atrophy of core clinical reasoning skills. Over reliance on AI dialogue systems negatively affects students’ critical cognitive capabilities, including decision making, critical thinking, and analytical reasoning (8, 27). A more pressing concern is that learners may fail to develop the essential skills of generating differential diagnoses and synthesising clinical information altogether. When AI tools provide rapid, ready−made lists of diagnostic possibilities and treatment options, students may never fully engage in the cognitive labor of constructing differentials from scratch or integrating disparate clinical data into coherent formulations. This represents a fundamental threat to competency development that is more consequential than simply avoiding cognitive discomfort. If residents and graduate trainees become accustomed to receiving rapid AI generated differentials or treatment suggestions, they may not develop the capacity for independent judgment that are hallmarks of competent psychiatrists. As educators, we have observed learners increasingly defaulting to AI generated answers rather than wrestling with clinical complexity. This pattern, if left unchecked, could produce a generation of clinicians who are proficient at retrieving information but ill-equipped to manage novel or complex presentations.

### 3.4 Dehumanization and erosion of therapeutic skills

Psychiatry is fundamentally a human encounter. Over reliance on AI for tasks traditionally requiring human interaction, even for practice purposes, risks normalizing a mechanical, scripted approach to patient communication. The therapeutic alliance, consistently shown to predict treatment outcomes across psychiatric conditions, cannot be modeled or taught through interactions with non-sentient systems (7, 28). Learners who spend excessive time practicing on AI avatars may struggle to read subtle nonverbal cues, respond to unexpected emotional expressions, or tolerate the discomfort of genuine therapeutic encounters. Research suggests that the externalization of internal experiences through generative AI in psychotherapy raises complex ethical questions about authenticity and the therapeutic relationship (29).

### 3.5 Ethical ambiguity and accountability gaps

While these questions have clear clinical implications, they are equally relevant to training. Trainees must understand the accountability structures that govern AI use before they enter independent practice, and training programs must establish clear policies to protect both learners and patients. When an AI chatbot provides a treatment suggestion that a trainee follows, leading to patient harm, who is accountable? The trainee? The supervising attending? The institution that approved the tool’s use? The developer? Current regulatory frameworks lack clarity on such questions. A systematic review by Dehbozorgi et al. (28) emphasized that while AI offers substantial potential to revolutionize mental health care through enhanced accessibility and personalized interventions, its integration must be guided by robust ethical frameworks. Until these accountability mechanisms are established, the use of GenAI in clinical training should remain under careful, case by case supervision (7).

## 4 Potential benefits of GenAI chatbots in psychiatric education

### 4.1 Enhanced access to information and learning efficiency

By acting as a powerful research and synthesis assistant, GenAI chatbots empower psychiatric learners to cut through information overload (30, 31). This frees up cognitive resources for higher order critical thinking and clinical application. These AI systems can process and synthesize vast amounts of medical literature, research findings, and clinical guidelines much faster than a human can. They provide learners with quick and comprehensive answers to their queries (15, 32). Research by Yim et al. (17) synthesized preliminary evidence on how GenAI assists, guides, and automates clinical service rendering, noting significant efficiency gains.

The ability of AI to provide information in multiple languages can also benefit international medical graduates or students for whom English is not their first language, thereby promoting inclusivity in learning (33).

### 4.2 Clinical skills simulation and practice

The development of robust clinical skills is a cornerstone of psychiatric training. GenAI chatbots present a promising tool for enhancing simulation-based learning. These AI systems can simulate patient encounters, allowing learners to practice psychiatric interviews, diagnostic formulation, and treatment planning in a controlled, low stakes environment (12, 34). Unlike standardized patients, AI chatbots can offer a vast range of patient personas with diverse symptomatology, cultural backgrounds, and communication styles. This exposes learners to a broader spectrum of clinical scenarios than might be encountered through traditional methods alone.

For example, learners can engage in simulated dialogues where they practice delivering a diagnosis, discussing treatment options, or responding to a patient in crisis. Research by Yin et al. (29) demonstrated that different AI driven chatbot feedback mechanisms can significantly influence learning outcomes and brain activity patterns. Moreover, Ajluni (12) found that AI can enhance psychiatric education by offering consistent, adaptable learning experiences that prepare trainees for real world complexities.

### 4.3 Support for clinical decision making and case-based learning

GenAI chatbots can offer valuable support for clinical decision making and enrich case-based learning (CBL) in psychiatric training. CBL is a cornerstone of medical education, and AI can significantly enhance its delivery and effectiveness (35). AI tools can generate a diverse array of detailed and nuanced case vignettes, covering a wide spectrum of psychiatric disorders, complexities, and patient demographics (4, 7, 28).

### 4.4 Addressing mental health needs and burnout among learners

Psychiatry trainees, especially at the graduate level, face significant stressors and are at a higher risk of burnout due to the emotionally demanding nature of their work (2). GenAI chatbots may play a role in addressing these mental health needs and promoting well-being (2, 7). These AI tools can offer a readily accessible, confidential, and non-judgmental platform for learners to discuss their stressors, anxieties, and emotional challenges (14, 15). However, we emphasize that AI chatbots should not replace traditional mental health support or professional supervision. Learners should always be encouraged to seek support from their supervisors, educators, or formal mental health services when experiencing distress. AI chatbots may serve as an adjunctive resource for basic psychoeducation and self-monitoring, but they are not a substitute for human professional support.

The 24/7 availability of AI chatbots means that support is always at hand. This is particularly beneficial during off hours or when traditional mental health services are not readily accessible (14). Some AI chatbots are designed to deliver elements of evidence based psychotherapies, such as cognitive behavioral therapy (CBT) or mindfulness exercises, which can help learners manage symptoms of anxiety or depression (2, 34). Cui et al. (36) found that an LLM based suicide intervention chatbot achieved high effectiveness in terms of emotional support, intervention efficacy, safety, privacy, and overall satisfaction. By providing an outlet for emotional expression and tools for self-care, AI chatbots can contribute to reducing feelings of isolation and improving overall mental well-being.

## 5 A framework for responsible integration

Given these risks, how should psychiatric educators proceed? We propose three interconnected principles.

### 5.1 Human oversight as an inviolable requirement

No AI generated output should be accepted without critical human evaluation (37). This means integrating AI into educational workflows in ways that require rather than replace learner judgment. For example, when AI generates a differential diagnosis list, learners should be required to justify the inclusion or exclusion of each item based on patient history and mental status examination. This turns AI output into a starting point for reasoning rather than a shortcut around it. As Ng et al. (18) emphasized, successful integration of GenAI in education requires strategies that encourage both teachers and students to embrace AI while maintaining critical oversight. To implement this in practice, we propose a tiered oversight model encompassing four levels: direct supervision, where learners discuss AI outputs with supervisors during routine teaching sessions; structured review, where supervisors periodically examine learners’ AI interaction logs; periodic audit, where training programs conduct systematic reviews of AI use across cohorts; and emergency override, where clear protocols are established for situations where AI generates potentially harmful recommendations (38).

The requirement to justify AI-generated outputs applies in structured educational settings, such as case-based learning sessions, supervisory meetings, and classroom discussions, where learners present clinical reasoning and are expected to explain their thought processes. This approach integrates critical evaluation into existing pedagogical practices without requiring surveillance of private study. Recent literature emphasizes that clinical supervisors must explicitly teach, assess, and model critical thinking when learners interact with AI (39). A structured framework such as DEFT−AI (Diagnosis, Evidence, Feedback, Teaching, and Recommendation for AI engagement) can be used by educators to scaffold critical thinking during AI interactions (40). These approaches complement the tiered oversight model described above.

### 5.2 AI literacy as a core curricular competency

We cannot simply tell students to “be careful” with AI. We must systematically teach them how to use it wisely (7). AI literacy curricula for psychiatric learners should include: (a) understanding how LLMs work, including their statistical rather than symbolic reasoning nature; (b) recognizing typical failure modes, including hallucinations, biases, and sycophancy; (c) practicing critical evaluation of AI outputs against authoritative sources; and (d) identifying situations where AI should not be used at all. Several recent reviews have emphasized that such training is urgently needed and currently lacking in most psychiatric training programs (2, 3, 7, 37).

AI literacy for psychiatric learners comprises four interconnected competency domains: foundational understanding (how LLMs function and their common failure modes), critical appraisal (evaluating AI outputs against authoritative sources), practical application (using AI tools effectively for tasks such as literature synthesis and case preparation), and ethical discernment (understanding when AI use is appropriate and how to maintain professional accountability). These competencies should be introduced progressively: medical students begin with foundational understanding; residents advance to practical application and ethical discernment; and practicing psychiatrists engage with governance and policy discussions. Pedagogically, we recommend case-based learning, interdisciplinary modules co-taught by clinicians and data scientists, and faculty development programs.

### 5.3 Institutional governance as a necessary safeguard

Individual vigilance by both educators and learners, while essential, is insufficient; institutions must establish governance mechanisms that complement individual efforts, including AI ethics committees, mandatory error reporting systems, regular policy reviews, and clear protocols for auditing AI tools (14, 41). These policies should address permissible use cases, data handling protocols, mandatory reporting of AI-related errors, and mechanisms for ongoing review as technology evolves. Research by Hipgrave et al. (14) highlighted the need for practitioner education and guidelines to support safe and effective AI integration in mental health care. A recent systematic review of AI mental health chatbots makes this point concrete. Of LLM−based chatbots, only 16% underwent clinical efficacy testing, with most remaining in early validation (42). The review proposed a three−tier evaluation framework—foundational bench testing, pilot feasibility testing, and clinical efficacy testing—to distinguish technical novelty from demonstrated clinical benefit (42). Adopting such a staged model in psychiatric education would ensure that learners encounter only chatbots that meet appropriate safety and efficacy standards before any patient−facing application.

Educators must remain alert to how their students are using AI and intervene when problematic patterns emerge. Learners must develop self-awareness about their own reliance on AI. However, individual vigilance alone is insufficient because it places the entire burden of responsible use on individuals without structural support. Institutions must therefore establish governance mechanisms that complement individual efforts, including: (a) an AI ethics committee comprising faculty, trainees, ethicists, and IT security experts; (b) mandatory error reporting systems; (c) regular policy reviews; and (d) clear protocols for auditing AI tools before introduction into educational or clinical settings (43).

## 6 Institutional recommendations

Based on the framework above, we offer six concrete recommendations for psychiatric training institutions. These are summarized in **Table 1**. Each recommendation carries specific implementation considerations. For policy and governance, institutions must move beyond generic AI policies to develop context-specific guidance for psychiatric education. For education and training, programs should not assume digital nativity equates to AI literacy; structured curricula are essential. For clinical integration, the key challenge is defining clear boundaries—what tasks may be delegated to AI and what tasks must remain exclusively human. For data governance, institutions must establish clear protocols for de-identification and secure data handling. For research and evaluation, funding agencies and institutions should prioritize studies that move beyond descriptive accounts to rigorous outcome evaluations. For transparency and accountability, institutions should publish their guidelines openly and review them annually, involving learners in the review process. For medical students, AI literacy should focus on foundational understanding and critical appraisal, emphasizing that AI should never substitute for clinical judgment. For residents, training should advance to practical application and ethical discernment, including integrating AI outputs into clinical reasoning while maintaining professional accountability. Supervisors should be trained to recognize different patterns of AI use at each level.

**Table 1:** Institutional recommendations for responsible GenAI integration in psychiatric education.

Recommendation category	Specific actions	Rationale
Policy and governance	Develop clear institutional usage policies; establish an AI ethics oversight committee	Ensure consistent, responsible use; address gaps in accountability
Education and training	Mandate AI literacy training for all learners and faculty; integrate critical evaluation skills into existing curricula	Equip trainees to use AI safely and effectively
Clinical integration	Require human in the loop protocols for any AI tool used in training contexts; prohibit unsupervised AI in patient facing assessments	Prevent over reliance and erosion of clinical judgment
Data governance	Vetting of all AI platforms for HIPAA/FERPA compliance; prohibit input of identifiable patient data into unsecured tools	Protect patient and learner confidentiality
Research and evaluation	Support rigorous studies on AI’s educational impact; fund research on bias mitigation and safety protocols	Generate evidence to inform best practices
Transparency and accountability	Publish institutional guidelines; establish clear liability frameworks for AI-related errors	Build trust and clarify responsibility

## 7 Future directions

The pace of GenAI development shows no sign of slowing. In five years, these tools will be more capable, more integrated into healthcare workflows, and more difficult to avoid. Continued research is essential to rigorously evaluate the efficacy and safety of AI chatbots in various educational and supportive roles. We need to understand their long-term impact on skill development and professional identity. We also need to develop effective strategies for mitigating risks such as bias and inaccuracy (28).

Concurrently, there is an urgent need for the development and refinement of comprehensive ethical frameworks that can guide the responsible use of AI in mental health and medical training (7). These frameworks must address novel ethical challenges, including data governance, algorithmic transparency, accountability for AI driven outcomes, and the preservation of humanistic care. Finally, regulatory bodies must evolve to keep pace with technological advancements, establishing clear standards for the validation, deployment, and monitoring of AI tools in healthcare and education.

The question is not whether AI will be used in psychiatric education but how. We need robust, methodologically sound research to evaluate AI’s impact on learning outcomes, skill development, and patient safety. We need professional bodies to develop national or international guidelines. And we need educators to embrace their role not as gatekeepers against technology but as guides who help learners navigate its complexities while preserving the humanistic core of psychiatry.

## 8 Conclusion

Generative AI offers genuine potential to enhance psychiatric education, but only if integrated with wisdom, humility, and clear safeguards. The path forward is neither enthusiastic adoption nor fearful prohibition. Rather, it is a deliberate, evidence informed approach that prioritizes human judgment, cultivates critical AI literacy, and establishes robust institutional governance. As psychiatric educators, our responsibility is to prepare learners not only to use these tools but to do so in ways that strengthen, rather than undermine, the clinical competencies and ethical commitments that define our profession. The ultimate goal is to harness the efficiency and innovation offered by generative AI while steadfastly protecting the core humanistic values fundamental to psychiatric practice and education.

## Data Availability

The original contributions presented in the study are included in the article/supplementary material. Further inquiries can be directed to the corresponding author.
